# Being helpful and being innovative: The role of psychological meaningfulness and positive affect

**DOI:** 10.3389/fpsyg.2022.1045845

**Published:** 2022-11-11

**Authors:** Yifei Shen, Zhenduo Zhang, Haoyang Song, Junwei Zheng, Qiong Bu

**Affiliations:** ^1^School of Economics and Management, Dalian University of Technology, Dalian, China; ^2^School of Information Management, Sun Yat-sen University, Guangzhou, China; ^3^Faculty of Civil Engineering and Mechanics, Kunming University of Science and Technology, Kunming, China; ^4^China Business Executives Academy, Dalian, China

**Keywords:** helping behavior, innovative behavior, psychological meaningfulness, positive affect, conservation of resources theory

## Abstract

The present study is developed based on conservation of resources theory (COR) to explore the underlying mechanism and boundary condition for the relationship between helping behavior and innovative behavior. To avoid the shortages of cross-sectional data, the present study collected two-wave and multi-source data. By collecting from 193 full-time Chinese workers and 68 supervisors at two separate time points, this study developed and examined a moderated mediation model using Mplus 7.0. The results show that helping behavior increases innovative behavior through enhancing positive affect, and psychological meaningfulness moderates the indirect relationship between helping behavior and innovative behavior through positive affect. In the condition of high psychological meaningfulness, helping behavior has a stronger indirect impact on innovative behavior through enhancing positive affect. This study enriches the literature on the outcomes of helping behavior. Moreover, this study provides several managerial implications to amplify the positive impact of helping behavior on innovative behavior. This study develops several strategies to enhance psychological meaningfulness and promote the benefits of helping behavior.

## Introduction

Helping behavior denotes voluntary assistance given to coworkers in order to accomplish goals or prevent problems (Yue et al., 2017). Given its positive influences on facilitating organizational effectiveness and team performance (Ehrhart et al., 2006), prior research has focused on the antecedents of helping behavior, such as diverse kinds of leadership and human resource management systems (Mossholder et al., 2011).

In recent times, several studies have explored the outcomes of helping behavior from an actor-centric perspective. For example, Gabriel et al. (2018) found that helping behavior caused actors to experience ego depletion daily. Although many studies have explored the psychological and behavioral outcomes of helping behavior, only a few have linked helping behavior to innovative behavior. To help coworkers cope with difficulties at work, employees need to integrate their knowledge to form a coping strategy (Bolino and Grant, 2016). Moreover, helping coworkers may aid them to cultivate positive emotions (Lin et al., 2017). These two resources are key roles in stimulating innovative behavior. In line with prior studies, this study proposes that helping behavior can be transmitted into innovative behavior. Getting insights into this research topic is important because employees are encouraged by their organization to help improve organizational effectiveness. Therefore, organizations should be mindful of the benefits and costs of helping behavior for helpers. The present study provides practical guidance to transform helping behavior into innovative behavior and leverage the management of helping behavior by examining this relationship.

Helping behavior has been regarded as an emotion regulation tool, which assists helpers to maintain positive emotional experiences (Lin et al., 2017). For example, Duan et al. (2019) found that helping behavior aids helpers to acquire positive affective experience. Affect is the “hot unit” that responds to helping behavior instantly. Prior studies have highlighted the “doing good, feeling good, and doing good” effect, which demonstrates how helping behavior promotes helpers’ positive affects, thereby encouraging helpers’ proactive behavior. For example, Lin et al. (2017) found that helping coworkers increases helpers’ positive affects and then promotes the emotional support they provide to their spouses. Due to the positive relationship between helping behavior and innovative behavior, this study proposes that positive affect plays a mediating role in the relationship between helping behavior and innovative behavior.

However, it is should be addressed that Lin et al. (2020) found a paradoxical result indicating that helping behavior could cause emotional exhaustion for the helpers. The potential explanation for these paradoxical research outcomes may be the omission of employees’ possessed job resources. Conservation of resources theory (COR) addresses the impacts of resources in facilitating employees’ in-role performance, coping with stress, and achievement of work goals (Halbesleben et al., 2014). To explore the boundary condition under which helping behavior is more or less effective in nurturing positive affect, this study introduces psychological meaningfulness as a moderator in the indirect relationship between helping behavior and innovative behavior through positive affect.

Psychological meaningfulness refers to the value of a work goal, judged in relation to an individual’s own ideals or standards (May et al., 2004). Psychological meaningfulness is an important job resource that exerts influence on the outcomes of helping behavior (Lin et al., 2020). Enhanced psychological meaningfulness makes employees believe that their investments of personal resources in helping others will be well reciprocated (Kahn, 1990; Lin et al., 2020). Although helping behavior can cause an extra emotional burden for helpers, psychological meaningfulness can work as a shield to helpers experiencing such stressful conditions through facilitating the recovery of resources (Ugwu and Onyishi, 2018). Thus, their helping behavior garners more positive emotional experiences, which can subsequently be beneficial for innovative behavior when psychological meaningfulness is high.

**FIGURE 1:**
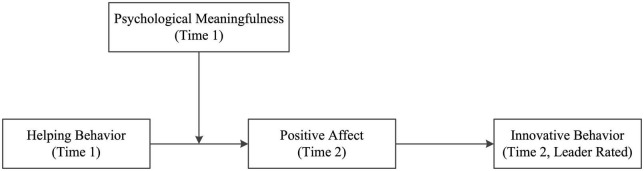
The conceptual model.

The basic tenet of COR theory, which is that individuals strive to obtain, retain, and protect job resources, has implications for understanding the outcomes of helping behavior. Previous studies have identified helping behavior as both resources-generating and resources-consuming, which shapes subsequent psychological states and behavioral responses. Thus, we developed a moderated mediation model (Figure 1), based on COR theory, to explain the ebbs and flows of resources triggered by helping behavior (Halbesleben et al., 2014).

We collected two-wave leader-subordinate dyadic data to test the conceptual model. In doing so, this study makes three contributions to the helping behavior literature and COR theory. First, this study extends our understanding of the outcomes of helping behavior by linking helping behavior to innovative behavior. Prior studies have mainly explored whether helping behavior impedes wellbeing or is transmitted into unethical behavior from an actor-centric perspective (Yam et al., 2017). However, the relationship between helping behavior and innovative behavior is not addressed, which makes the benefits of helping behavior less known. When lending hands to coworkers, helpers are motivated to integrate possessed knowledge and cultivate positive affective experiences through their interactions with coworkers, which are important to enhance innovative behavior (Bolino and Grant, 2016). This study attempts to explore the innovative outcomes of helping behavior, thereby providing a comprehensive insight into the advantages of helping behavior for helpers.

Second, this study uncovers the mediating role of positive affect in the relationship between helping behavior and innovative behavior. The benefits of helping behavior for helpers have been addressed by ample studies, especially for its emotional benefits (Shah et al., 2018; Lin et al., 2020). From the “doing good, feeling good” perspective, this study identifies positive affect as the key resource linking helping behavior to innovative behavior. Positive affect broadens helpers’ behavioral and thinking repertories, which is beneficial for their innovative performance. We specify the underlying emotional path through which helping behavior is transmitted into innovative behavior, thereby contributing to the mechanisms explaining how helping behavior impacts following behaviors.

Third, this study adopts psychological meaningfulness as a moderator, providing a potential explanation for the paradoxical emotional outcomes of helping behavior and extending the scope of the COR theory. Prior studies have provided two different impacts of helping behavior on positive affective experiences. Lin et al. (2017) examined the positive relationship between helping behavior and positive affects. However, Lin et al. (2020) found that helping behavior leads to emotional exhaustion. One of the explanations for these paradoxical outcomes is the neglection of helpers’ possessed resources. From the COR perspective, this study adopts psychological meaningfulness, a key resource for helpers, as the moderator specifying the boundary condition that impacts the relationship between helping behavior and positive affects.

## Hypothesis development

### Helping behavior and innovative behavior

For helpers, the aim of helping behavior is to aid those who seek help in coping with difficulties at work (Yue et al., 2017). To achieve this aim, helpers need to evaluate prior unsuccessful attempts to solve the problem (Yue et al., 2017). This means that helpers and helping seekers confront the same challenge in that they are both exposed to different aspects of the challenging task and diverse information when making problem-solving efforts (Mueller and Kamdar, 2011). Based on this, Perlow and Weeks (2002) proposed that helping behavior is a beneficial opportunity for helpers to improve their skills and fill their knowledge gaps. Helpers need to integrate the knowledge they possess with problem-related information to improve their critical thinking capabilities and to develop more creative ways to solve problems (Yánez Morales et al., 2020). Through helping others, helpers may acquire new knowledge and apply this knowledge in both familiar and unfamiliar situations (Li and Liao, 2017). Through this process, helpers will gain deep insights into the difficulties they have faced and form their cognitive schema by integrating their new knowledge, which is beneficial for developing innovative ideas.

During the problem-solving procedure, helpers and helping seekers may have a beneficial interaction in which they exchange information or necessary resources to overcome their difficulties (Lin et al., 2020). In doing so, it is a necessary condition for helpers to acquire valuable resources to develop novel ideas (Zhang et al., 2022). When successfully helping coworkers, helpers usually obtain gratitude from them. Helping behavior also aids helpers in cultivating a high reputation at work and establishing high-quality social relationships with their coworkers; these are key components of social capital, which are sources of positive emotions (Zhang et al., 2020). Fredrickson (2004) suggested that positive affect enhances individuals’ cognitive flexibility and promotes their confidence in engaging in innovative behavior. Combining these arguments, this study puts forward the following hypothesis:

*H1: Helping behavior is positively associated with innovative behavior.*

### Mediating role of positive affect

Positive affect reflects the extent to which a person feels enthusiastic, alert, and active. With high positive affect, individuals are in a state of high energy, full concentration, and pleasurable engagement (Watson et al., 1988). Prior studies have found that helping is a positive and voluntary interpersonal activity that has the potential to enhance helpers’ positive affect due to its generation of psychological resources (Lin et al., 2017). Helping behavior is an affiliative endeavor facilitating social cohesiveness by which employees build reciprocal ties with coworkers (Koopman et al., 2016). Furthermore, helping behavior enhances core-self evaluations and helpers usually receive gratitude from recipients because helpers solve work-related problems for them (Lee et al., 2019). Employees thus gain personal emotional resources and have positive affective experiences after helping coworkers.

COR theory suggests that individuals invest job resources as a means to obtain additional resources, potentially creating virtuous cycles. Both theoretical and empirical studies have found that helping behavior facilitates the cultivation of positive affect from an actor-centric perspective. Bolino and Grant (2016) suggest a positive relationship between helping behavior and positive affect in their review. Lin et al. (2017) found that helping behavior nurtures helpers’ positive affect on a daily basis, thus confirming the “doing good-feeling good” effect. Laboratory studies have shown that simple helping behavior enhances individuals’ positive affect (Yinon and Landau, 1987). Therefore, the following hypothesis was proposed:

*H2a: Helping behavior is positively associated with positive affect.*

We propose that work-related positive affect plays a critical role in stimulating the innovative behavior of helpers. COR theory suggests that positive affect is a valuable resource that broadens individuals’ behavioral repertories and attention (Fredrickson and Joiner, 2018). Therefore, positive affect facilitates helpers’ cognitive flexibility and is associated with a growth mindset (Williamson et al., 2019), thereby boosting divergent thinking and innovative behavior (Yuan et al., 2018).

Positive affect signals an increase in the possibility of achieving favorable outcomes and usually elicits more additional positive affect, which thus further favors innovative performance (Pillay et al., 2020). Prior studies have provided fruitful evidence for the positive relationship between positive affect and innovative behavior. De Rooij and Vromans (2020) used spontaneous eye blink rate to explore the relationship between positive affect and divergent thinking, one of the two thinking processes of creative idea generation. de Buisonjé et al. (2017) indicated that positive affect facilitates creative idea selection.

Moreover, Koopman et al. (2016) posit that the affective boost associated with helping behavior is consequentially turned into affective commitment and job satisfaction. Innovation requires employees to devote certain job resources, such as time and energy (Kwon and Kim, 2020). Both affective commitment and job satisfaction provide helpers with the senses of belonging, stability, and security, which allow helpers to engage in innovative behavior with minimal expenditure of energies and enhance their willingness to devote time to applying novel solutions to improve organizational effectiveness (Lapointe et al., 2011; Gillet et al., 2018). Thus, the following hypothesis was proposed:

*H2b: Positive affect is positively associated with innovative behavior.*

As aforementioned, the “doing good-feeling good” effect has been confirmed by theoretical and empirical research (Lin et al., 2017). The cultivation of appreciation and anticipated reciprocal support from helped colleagues simulate helpers’ positive experiences (Koopman et al., 2016; Lin et al., 2020). Consequently, enhanced positive affect motivates helpers to devote time and energies to engage in innovative behavior. Moreover, positive affect releases cognitive resources and creative thinking, thereby facilitating innovative behavior (Lapointe et al., 2011). Therefore, the following hypothesis was proposed:

*H2c: Positive affect mediates the positive relationship between helping behavior and innovative behavior.*

### Moderating role of psychological meaningfulness

Psychological meaningfulness is defined as the significance a person attaches to an object, event, or situation (Ugwu and Onyishi, 2018). The organizational behavior literature uses meaningfulness of work to indicate the value of a work goal or purpose, judged in relation to an individual’s own ideals or standards (May et al., 2004). At work, psychological meaningfulness is beneficial to both employees and organizations. Employees are motivated to seek meaning in daily work (Aguinis and Glavas, 2019). Employees will disengage from their work in the absence of psychological meaningfulness (May et al., 2004). Psychological meaningfulness is a core psychological process connecting perceptions of the work environment with psychological experience (Chaudhary, 2022). It acts as a motivational pathway in which values and purposes derived from the work context are transformed into a fulfilling and positive personalized experience (Mostafa and El-Motalib, 2020).

COR theory suggests the important role of personal resources in shaping individuals’ resource conservation and generation processes (Halbesleben et al., 2014). COR theory posits that when individuals regard their job situation as favorable and appreciated, beneficial psychological energies motivate them to undertake helping behavior to contribute to their coworkers’ wellbeing, and also to reward themselves with desirable affective experiences and anticipated reciprocation (Halbesleben et al., 2014). Furthermore, aligning with the resource gain spirals tenet of COR theory, employees are more likely to cultivate positive energies from helping behavior when they have access to complementary resources to undermine the effects of potential loss of psychological resources (Zhang et al., 2020). De Clercq et al. (2019) suggested that psychological meaningfulness captures the extent to which employees deem their work to be important. Psychological meaningfulness enhances helpers’ favorable feelings about their job situation and provides them with complementary psychological resources, which invigorates the relationship between helping behavior and favorable emotional experiences. Therefore, the present study adopts psychological meaningfulness, an important job resource, as a moderator in the relationship between helping behavior and positive affect.

Those with high psychological meaningfulness will hold a belief that their investments of job resources in helping coworkers will be well reciprocated, which inhibits their worries about job losses (Kahn and Heaphy, 2013). As well, the sense of meaningfulness enables individuals to overcome job demands and boost the positive affective experiences caused by helping behavior (Ugwu and Onyishi, 2018). In contrast, when helpers have lower levels of psychological meaningfulness, they will expect that there will be few gains in or returns from their investments of personal resources in helping others (Kahn and Heaphy, 2013). This feeling of resource loss will decrease the positive affective experiences induced by helping behavior. Moreover, those with a lower level of psychological meaningfulness are more vulnerable to the job demands caused by helping behavior and have a decreased likelihood that they will experience positive affect. The following hypothesis was thus proposed:

*H3: Psychological meaningfulness moderates the relationship between helping behavior and positive affect, such that in the condition of higher psychological meaningfulness, the association between helping behavior and positive affect will be stronger.*

As aforementioned, positive affect mediates the relationship between helping behavior and innovative behavior. COR theory suggests that individuals who start with more job resources are less impacted by the uncertainty caused by the loss of job resources (Halbesleben et al., 2014). Psychological meaningfulness has been used in research based on COR theory, and it is viewed as a valuable resource leveraging the sense of fulfillment and positive energies (De Clercq et al., 2019). Therefore, those with high psychological meaningfulness are less sensitive to the loss of job resources caused by helping behavior. Furthermore, psychological meaningfulness is associated with achievements of resource gain spirals. For example, Mostafa and El-Motalib (2020) encourages the investment of positive energies into one’s work, which enables helpers to better manage and integrate their social relationships, thereby improving their wellbeing. High psychological meaningfulness enables helpers to reap more positive affect through helping coworkers to deal with difficulties encountered at work and then stimulates more innovative behavior. In contrast, low psychological meaningfulness makes helpers more likely to experience emotional exhaustion due to the interrupted work routine and impedes the work progress triggered by helping behavior without complementary job resources. Therefore, low psychological meaningfulness decreases the likelihood that helpers will obtain positive affect through helping coworkers, thereby inhibiting the performance of innovative behavior at work. Therefore, the following hypothesis was proposed:

*H4: Psychological meaningfulness moderates the indirect relationship between helping behavior and innovative behavior through positive affect, such that in the condition of higher psychological meaningfulness, this indirect relationship is stronger than in the condition of lower psychological meaningfulness.*

## Materials and methods

### Participants and procedure

Data were collected from employees in a construction enterprise in Beijing, China. Before data collection began, human resource managers sent an announcement to the group leaders, explaining the research purpose and research procedure, and asked for their willingness to engage in the survey. Ultimately, 73 group leaders gave responses to the announcements. The website links for the survey were sent to the group leaders and they communicated the website links to their employees.

Prior psychological studies have adopted cross-sectional data to collect respondents’ attitudes, beliefs, and perceptions of the situations reported by the same person at the same time points. Consequently, there is a possibility that common method variance (CMV) has artifactually inflated the observed correlations between these types of variables. Feldman and Lynch (1988) further suggested that behavioral self-reports could be significantly correlated with job dimensions that are completely meaningless to the respondents if they are required to assess their own performance (i.e., job performance and innovative performance) and then provide ratings of job characteristics and psychological states related to such performance. Podsakoff et al. (2003) has suggested that CMV may result in inflated correlations between variables collected through cross-sectional data and also developed remedies for such bias. The first is to collect data from different time points and the second is to collect data from different sources (Podsakoff et al., 2003). Our research mainly explores the relationship between helping behavior, positive affect, psychological meaningfulness, and innovative behavior. The variables are employees’ psychological attitudes and behaviors. If we were to use a cross-sectional design, the correlations between these variables may be exaggerated, yielding results that are ultimately meaningless (Feldman and Lynch, 1988). For these reasons, we collected survey data at two time points in leader-subordinate dyads to control CMV and enhance the reliability of the results. At Time 1, participants were asked to report their levels of helping behavior and psychological meaningfulness. At Time 2, a month after Time 1, employees were asked to report their positive affect, and the group leaders were asked to report employees’ innovative behavior. The leader-reported innovative behavior may enhance the objectivity of the ratings of innovative performance and the two-time point lagged design may decrease the bias caused by CMV (Leung et al., 2011; Peng et al., 2019). In both surveys, employees were asked to report their demographic information, including code, age, education, gender, and position in their organization, which were used to match the data.

Surveys were returned by 227 employees at time 1, and by 212 at time 2 (response rate 93.39%). At time 2, 68 of 73 group leaders finished their questionnaires, giving a response rate of 93.15%. Among the 212 participants, there were 17 employees whose leaders failed to finish the questionnaire, and 2 questionnaires were partially finished. Ultimately, 193 employees nested in 68 group leaders finished the questionnaire, giving an effective response rate of 84.64%. The participants worked in the management department (24.8%), information technology department (15.4%), design department (32.7%), and the frontline department (27.1%). 50.8% of the participants were male; 19.2% of the participants had a college certificate or below, and 17.6% had a master’s degree or above. Participants’ average age was 32.15 years (±6.25).

### Measures

We followed Brislin’s (1980) suggestion to conduct a translation-back translation procedure to ensure the accuracy of the measures which were originally developed in English. The measurements used in this study are listed in Appendix 1.

#### Helping behavior

Three items developed by Yue et al. (2017) were used to measure helping behavior at time 1. A sample item is, “I help my colleagues when it is clear their workload is too high.” A five-point Likert scale was used to measure how frequently employees engaged in helping behavior in the last month, with 1 = *never* and 5 = *always.*

#### Psychological meaningfulness

Six items developed by May et al. (2004) were used to measure psychological meaningfulness as rated by employees. A sample item is, “The work I do on this job is very important.” A five-point Likert scale was used, ranging from 1 = *strongly disagree* to 5 = *strongly agree*.

#### Positive affect

The short form of the PANAS scale was used to measure positive affect at time 2 (Thompson, 2007). Employees were asked to report how frequently they felt determined, attentive, alert, inspired, and active in the last month on a five-point Likert scale with 1 = *very slightly* and 5 = *extremely*.

#### Innovative behavior

The six-item innovative behavior scale developed by Scott and Bruce (1994) was used in the survey and completed by group leaders at time 2. A sample item is, “This worker generates creative ideas.” A five-point Likert scale was used ranging from 1 = *strongly disagree* to 5 = *strongly agree*.

#### Control variables

Considering the influences of gender, age and education on innovative behavior (Newman et al., 2018; Luu, 2019), this study controlled them in the structural equation modeling analysis and regression analysis.

## Results

### Analytical strategy

Given the nested structure of the innovative behavior ratings [i.e., 193 subordinates and 68 group leaders, ICC(1) = 0.51], the nested-equation path analytic approach was used to analyze the non-independence data (Wu et al., 2016). The “Type = Complex” and “Estimator = MLR” settings were used in Mplus 7.0. This approach was appropriate for this study because this study is concerned with non-independence data structures with data at the employee level (Zheng et al., 2021). After completing the nested-equation path analysis, the bootstrapping test was used to examine the robustness of the results.

### Confirmatory factor analysis

We examined the hypothesized measurement model with three factors: helping behavior, psychological meaningfulness, and positive affect. Because innovative behavior was rated by group leaders, it was not adopted in the confirmatory factor analysis (Laulié et al., 2021). The results in Table 1 showed that the three-factor model has a better fit (χ^2^ = 128.40, df = 73, SRMR = 0.05; CFI = 0.97, TLI = 0.97, RMSEA = 0.06) than other models (△χ^2^ ≥ 94.10), indicating the acceptable discriminant validity of the research variables.

**TABLE 1:** Results of confirmatory factor analysis.

Model	Variables	χ^2^	df	χ^2^/df	Δχ^2^	SRMR	CFI	TLI	RMSEA
Three-factor model	HB, PM, PA	128.40	73	1.76		0.05	0.97	0.97	0.06
Two-factor model	HB + PM, PA	305.30	75	4.07	176.90**	0.10	0.89	0.87	0.13
Two-factor model	HB, PM + PA	222.50	75	2.97	94.10**	0.07	0.93	0.92	0.10
Two-factor model	HB + PA, PM	266.83	75	3.56	138.43**	0.08	0.91	0.89	0.12
One-factor model	HB + PM + PA	384.11	76	5.05	255.71**	0.11	0.86	0.83	0.15

**p < 0.01; *N* = 193. HB, Helping Behavior; PM, Psychological Meaningfulness; PA, Positive Affect.

### Hypothesis tests

The means (M), standard deviations (SD), composite reliabilities (CR), average variances extracted (AVE), and correlations of all variables are shown in Table 2.

**TABLE 2:** Means, standard deviations, CR, AVE, and correlation analysis.

	AVE	CR	Mean	SD	1	2	3	4	5	6	7
1. Gender			1.49	0.50							
2. Age			32.15	6.25	0.03						
3. Education			1.98	0.61	0.21**	0.05					
4. Innovative behavior	0.64	0.91	3.86	0.80	0.00	0.05	−0.04	(0.91)			
5. Helping behavior	0.58	0.81	3.91	0.62	0.05	0.11	−0.02	0.21**	(0.80)		
6. Positive affect	0.53	0.84	3.80	0.60	−0.12	−0.02	0.01	0.22**	0.38**	(0.77)	
7. Psychological meaningfulness	0.76	0.95	4.06	0.69	−0.11	−0.01	0.01	0.12	0.31**	0.71**	(0.95)

**p < 0.01; *N* = 193. Values in the parentheses are the Cronbach’s alpha.

Given the nested nature of the data, we first analyzed the data using the “Type = Complex” and “Estimator = MLR” settings in Mplus 7.0. To test the mediating role of positive affect, we followed the procedure proposed by Kenny (2008). First, innovative behavior was regressed on helping behavior. Second, positive affect was regressed on helping behavior. Third, innovative behavior was regressed on helping behavior and positive affect simultaneously. If helping behavior and positive affect are both significant, but the significance of the helping behavior decreases, this implies that the influence of helping behavior on innovative behavior is partially mediated by positive affect. If helping behavior becomes not significant while positive affect is significant, the influence of helping behavior on innovative behavior is fully mediated by positive affect.

**FIGURE 2:**
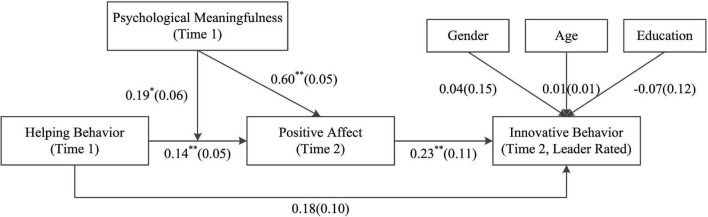
Results of nested-equation path analysis. Parameters are unstandardized; Values in the parentheses are standard errors; *N* = 193; *p < 0.05; **p < 0.01.

The results in Table 3 indicate that helping behavior is positively associated with innovative behavior (Model 5, *B* = 0.26, *SE* = 0.06, *p* < 0.01), supporting H1. Helping behavior is positively related to positive affect (Model 2, *B* = 0.38, *SE* = 0.07, *p* < 0.01), supporting H2a. When innovative behavior is regressed on helping behavior and positive affect simultaneously, helping behavior is not significant (Model 6, *B* = 0.18, *SE* = 0.10, n.s.), whereas positive affect is still significant (Model 6, *B* = 0.23, *SE* = 0.11, *p* < 0.05). These results support H2b and H2c. The results also indicate that positive affect may play as a full mediating role in the relationship between helping behavior and innovative behavior. To test the whole conceptual model, nested-equation path analysis is used to test the hypotheses, and the results are depicted in Figure 2.

**TABLE 3:** Results of hierarchical regression analysis.

	Positive affect						Innovative behavior					
	Model 1		Model 2		Model 3		Model 4		Model 5		Model 6	
	B	SE	B	SE	B	SE	B	SE	B	SE	B	SE
Gender	0.00	0.01	−0.01	0.01	0.00	0.00	0.01	0.01	0.00	0.01	0.01	0.01
Age	0.04	0.08	0.06	0.07	0.04	0.06	−0.06	0.12	−0.05	0.13	−0.07	0.12
Education	−0.15	0.10	−0.18	0.09	−0.07	0.06	0.02	0.14	0.00	0.14	0.04	0.15
Helping behavior			0.38**	0.07	0.15**	0.05			0.26**	0.10	0.18	0.10
Psychological meaningfulness					0.59**	0.05						
Helping behavior × psychological meaningfulness					0.19**	0.06						
Positive affect											0.23**	0.11
−2LL	−174.78		−158.49		−98.32		−228.91		−224.79		−222.24	
AIC	359.55		328.98		212.63		467.81		461.59		458.47	
BIC	360.03		329.55		238.74		484.13		462.16		481.31	

*p < 0.05, **p < 0.01; N = 193.

To further explore the mediating role of positive affect in the relationship between helping behavior and innovative behavior, a bootstrapping test is conducted, and the results are presented in Table 4. The results of the bootstrapping test indicate that the indirect relationship between helping behavior and innovative behavior through positive affect is significant [Table 4, Effect = 0.03, *SE* = 0.02, 95% CI = (0.01, 0.07)]. Several studies have suggested that this may be due to the “feeling good, doing good” effect rather than the “doing good, feeling good” effect. Therefore, we recalculate the alternative model about the indirect relationship between positive affect and innovative behavior through helping behavior. The indirect relationship is not significant [Effect = 0.07, *SE* = 0.04, 95% CI = (−0.01, 0.15)]. The insignificant alternative model supports H2c to a certain degree.

**TABLE 4:** Results of bootstrapping test.

	Effect	SE	95% CI	
			95% LL	95% UL
**Moderating effect of psychological meaningfulness**				
Low psychological meaningfulness (M − SD)	−0.04	0.07	−0.19	0.10
High psychological meaningfulness (M + SD)	0.32	0.08	0.17	0.47
Difference	0.36	0.11	0.14	0.59
**Mediation effect**				
Direct effect	0.18	0.10	−0.01	0.38
Indirect effect	0.03	0.02	0.01	0.07
**Moderated multiple mediation effect**				
Low psychological meaningfulness (M − SD)	−0.01	0.02	−0.05	0.03
High psychological meaningfulness (M + SD)	0.07	0.04	0.01	0.16
Difference	0.08	0.05	0.01	0.19

Bootstrapping = 20,000; CI, Confidence Interval; LL, Lower Level; UL, Upper Level.

**FIGURE 3:**
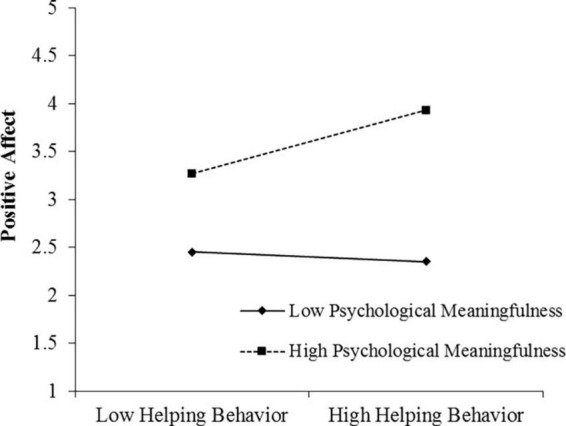
Results of moderating role of psychological meaningfulness in the relationship between helping behavior and positive affect.

In Table 3, the interactive item of helping behavior with psychological meaningfulness is positively associated with positive affect (Model 3, *B* = 0.09, *SE* = 0.06, *p* < 0.01), supporting H3. To further explore the moderating role of psychological meaningfulness in the relationship between helping behavior and positive affect, a simple slope test is conducted, and the results are presented in Table 4. When psychological meaningfulness is high (+1 *SD*), the relationship between helping behavior and positive affect is significant [Effect = 0.32, *SE* = 0.08, 95% CI = (0.17, 0.47)]. When psychological meaningfulness is low (−1 *SD*), the impact of helping behavior on positive affect is not significant [Effect = 0.18, *SE* = 0.10, 95% CI = (−0.01, 0.38)]. The difference in the two slopes is also significant [Effect = 0.36, *SE* = 0.11, 95% CI = (0.14, 0.59)]. The moderating effect of psychological meaningfulness is depicted in Figure 3, supporting H3.

Finally, this study tested the moderated mediation model by using a bootstrapping test. In the condition of high psychological meaningfulness (+1 *SD*), the indirect relationship between helping behavior and innovative behavior through positive affect is significant [Effect = 0.07, *SE* = 0.04, 95% CI = (0.01, 0.16)]. In the condition of low psychological meaningfulness (−1 *SD*), the indirect relationship is not significant [Effect = −0.01, *SE* = 0.02, 95% CI = (−0.05, 0.03)]. The difference between these two slopes is significant [Effect = 0.08, *SE* = 0.05, 95% CI = (0.01, 0.19)]. H4 is thus supported.

## Discussion

### Theoretical implications

The present study found an indirect relationship between helping behavior and innovative behavior through positive affect. This indirect relationship is moderated by psychological meaningfulness, and it is significant only in the condition of high psychological meaningfulness. This research thus provides several theoretical implications to the present research concerning helping behavior and COR theory.

First, this study extends our knowledge of the outcomes of helping behavior by exploring the relationship between helping behavior and innovative behavior. Although prior studies have explored both the long- and short-term outcomes of helping behavior (Koopman et al., 2020), less attention has been paid to the impact of helping behavior on the helpers’ innovative behavior. The omission of this impact leads to a lack of insights into the positive influence of helping behavior on employees’ growth in organizations. Prior studies have linked innovative behavior to employees’ career growth at work (Sue-Chan and Hempel, 2016). Helping behavior is a process in which individuals integrate their possessed knowledge and consistently interact with their coworkers (Bolino and Grant, 2016). The present study finds that helpers acquire positive emotional resources in this process, thereby stimulating innovative behavior. Establishing the relationship between helping behavior and innovative behavior improves our understanding of the benefits of helping behavior.

Second, this study uncovers the underlying emotional path through which helping behavior facilitates innovative behavior. Prior studies have demonstrated the positive emotional outcomes of helping behavior at both the within- and between-person levels. For example, Lin et al. (2017) found that daily helping behavior cultivates helpers’ positive affect. Prior studies have also found that helping behavior facilitates employees’ acquisition of positive emotional resources in the long run (Bolino and Grant, 2016; Duan et al., 2019). Prior studies have found that positive affect enhances individuals’ cognitive flexibility and divergent thinking, thereby facilitating innovative behavior (Williamson et al., 2019). From the “doing good, feeling good” perspective, helpers promote their own positive affect by cultivating gratitude and core self-evaluations from the coworkers they have helped to overcome difficulties at work (Lin et al., 2017). These positive affective experiences broaden their behavioral and thinking repertories, which are advantageous for improving their innovative behavior (Fredrickson, 2004). Moreover, this study found that the relationship between helping behavior and innovative behavior is fully mediated by positive affect. Research on links between helping behavior and other forms of behavior has mainly aimed at uncovering the underlying mechanism linking the two. For example, Gabriel et al. (2018) explored the relationship between helping behavior and political acts at the episode level. In their study, they hypothesized that the relationship between helping behavior and political acts is fully mediated by ego depletion. The basic tenet of this line of research is that one kind of behavior leads to changes in psychological states and then results in the other kind of behavior (Loi et al., 2020). This study contributes to this line of research by exploring the indirect relationship between helping behavior and innovative behavior through positive affect.

Third, the present study unveils the boundary condition under which helping behavior impacts innovative behavior through positive affect by exploring the moderating role of psychological meaningfulness. COR theory posits a role for personal possessed resources in shaping individuals’ resource conservation and generation process (Halbesleben et al., 2014). Although the majority of research uses the “doing good-feeling good” effect to explain the positive relationship between helping behavior and positive affect, Lin et al. (2020) also found that helping behavior can lead to emotional exhaustion. Based on COR theory, it is assumed that whether helping behavior acquires or depletes emotional resources depends on helpers’ possessed resources. Psychological meaningfulness has been viewed in COR theory as a buffer in the relationship between job demands and emotional reactions (Mostafa and El-Motalib, 2020). Meaningfulness not only enhances helpers’ belief in the reciprocity of investing resources to help their coworkers but also gives them resources to cope with the job demands caused by helping behavior to achieve a boost in affect (Tims et al., 2016). Furthermore, previous studies have suggested that psychological meaningfulness are the both outcome and the antecedent of helping behavior (Lin et al., 2020). Extending this line of research, the current study finds that psychological meaningfulness also shapes the emotional outcomes of helping behavior, which ensures the positive mechanism through which helping behavior is transformed into innovative behavior.

### Practical implications

This study also provides several practical implications for practitioners. This study finds that helping behavior is an inducement for positive affect and innovative behavior. Therefore, organizations should adopt the necessary strategies to motivate employees’ helping behavior. For example, Zhang et al. (2020) suggest that helping behavior can trickle down from leaders to employees. However, it should be noted that helping behavior may interrupt helpers’ work progress and increase their workload (Koopman et al., 2016). Thus, organizations should encourage employees to help colleagues strategically to minimize the disadvantages of helping behavior.

Psychological meaningfulness is also regarded as an amplifier in the indirect relationship between helping behavior and innovative behavior through positive affect. The results indicate the indirect emotional path emerges only in the condition of high psychological meaningfulness. Helpers can cultivate higher levels of positive affect through increasing psychological meaningfulness. Therefore, practitioners need to emphasize stimulating helpers’ psychological meaningfulness. To achieve this, organizations should redesign jobs to allow for employees’ decisions that enhance their impacts on organizations (May et al., 2004). As well, organizations should enable employees to develop deeper social connections with colleagues, allowing employees to understand their impacts on others (Lin et al., 2020).

### Limitations and future directions

This research has several limitations that could provide starting points for future research. First, the causal relationship between the focal variables cannot be inferred in this study. Although a two-wave research design was adopted, we did not manipulate the independent variable (i.e., helping behavior), which makes it difficult to infer a causal relationship between helping behavior and innovative behavior. Future research may use an experimental design or a cross-lagged design to make solid conclusions about the relationship between helping behavior and innovative behavior.

Second, CMV cannot be ruled out completely. We collected data at two distinct time points, rated by different subjects (i.e., innovative behavior was rated by supervisors), which can decrease the influence of CMV. However, psychological meaningfulness and helping behavior were measured at time 1 and rated by employees which could raise potential CMV bias. Future research could use coworker-rated or supervisor-rated helping behavior to rule out CMV completely.

Third, an alternative cognitive mechanism linking helping behavior and innovative behavior should be further explored. Bolino and Grant (2016) suggested that helping behavior enhances helpers’ personal cognitive information processing capability, which is a critical antecedent to innovative behavior. This study uncovered the emotional path by which helping behavior enhances innovative behavior, by exploring the mediating role of helping behavior. Future research should further investigate cognitive information processing capability and its relationship to the association between helping behavior and innovative behavior.

Finally, this research was conducted in a Chinese context, and this influences the external validity of our findings. Prior studies have found a relationship between collectivist culture and helping behavior (Alkhadher et al., 2020). Due to the prevalence of collectivist culture in Chinese enterprises, the costs and benefits of helping behavior may vary between China and western countries. Future research may conduct a cross-cultural study to compare the differences in the relationship between helping behavior and innovative behavior.

## Conclusion

By using a two-wave multi-source research design, this study collected data from 193 leader-supervisor dyads. We adopted nested-equation path analysis to analyze the data and test the conceptual model. The results showed that helping behavior enhances helpers’ positive affect, thereby facilitating innovative behavior. Furthermore, this indirect relationship is amplified by psychological meaningfulness, such that this indirect relationship is significant in the condition of high psychological meaningfulness. This research was conducted within the framework of COR theory. This study extended our understanding of the outcomes of helping behavior, and unveiled the emotional mechanism through which helping behavior can be transformed into innovative behavior. Moreover, this study contributes to COR theory by exploring the moderating role of psychological meaningfulness, which provides new insight into the costs and benefits of helping behavior.

## Data availability statement

The raw data supporting the conclusions of this article will be made available by the authors, without undue reservation.

## Author contributions

All authors listed have made a substantial, direct, and intellectual contribution to the work, and approved it for publication.

## Appendix 1

### Measurement in the study

1. Helping Behavior (Time 1, Yue et al., 2017)

HB1. I help other employees when it is clear their workload is too high.

HB2. I lend a helping hand to coworkers when needed.

HB3. I willingly assist other employees in meeting their job requirements.

2. Psychological Meaningfulness (Time 1, May et al., 2004)

PM1. The work I do on this job is very important to me.

PM2. My job activities are personally meaningful to me.

PM3. The work I do on this job is worthwhile.

PM4. My job activities are significant to me.

PM5. The work I do on this job is meaningful to me.

PM6. I feel that the work I do on my job is valuable.

3. Positive Affect (Time 2, Thompson, 2007)

PA1. I felt determined in the past month.

PA2. I felt attentive in the past month.

PA3. I felt inspired in the past month.

PA4. I felt alert in the past month.

PA5. I felt active in the past month.

4. Innovative Behavior (Time 2, Scott and Bruce, 1994)

IB1. The employee searches out new technologies, processes, techniques, and/or product ideas.

IB2. The employee generates creative ideas.

IB3. The employee promotes and champions ideas to others.

IB4. The employee investigates and secures funds needed to implement new ideas.

IB5. The employee develops adequate plans and schedules for the implementation of new ideas.

IB6. The employee is innovative.
